# Silencing without clearing: pharmacological modulation of the SASP in aging and disease

**DOI:** 10.3389/fphar.2026.1911529

**Published:** 2026-10-12

**Authors:** Lukas Fresser, Zofia Chrienova, Jan Gebrt, Patrik Oleksak, Kamil Kuca, Eugenie Nepovimova

**Affiliations:** 1 Department of Chemistry, Faculty of Science, University of Hradec Kralove, Hradec Kralove, Czechia; 2 Biomedical Research Centre, University Hospital Hradec Kralove, Hradec Kralove, Czechia; 3 Faculty of Informatics and Management, Center for Basic and Applied Research, University of Hradec Kralove, Hradec Kralove, Czechia; 4 Center of Advanced Innovation Technologies, VSB-Technical University of Ostrava, Ostrava-Poruba, Czechia

**Keywords:** age-related disease, aging, cellular senescence, SASP, senomorphics

## Abstract

The global rise in life expectancy has led to an increasing prevalence of age-related diseases, including neurodegenerative disorders, cancer, type 2 diabetes mellitus, and cardiovascular conditions, placing substantial societal and economic burdens on healthcare systems worldwide. A key mechanism underlying both aging and these pathologies is cellular senescence (CS), now recognized as one of the 12 hallmarks of aging. Senescent cells exert their effects largely through the senescence-associated secretory phenotype (SASP), a complex pro-inflammatory secretome that disrupts tissue homeostasis in neighbouring cells and actively promotes disease progression. This review first describes the biology of CS and the SASP in the context of aging and age-related pathologies, with emphasis on key regulatory pathways. We then provide a comprehensive overview of current senomorphic agents, i.e., compounds that modulate the SASP without eliminating senescent cells, covering their mechanisms of action, the signaling pathways they target, and the translational challenges they face. A particular focus is placed on the context-dependent dual role of the SASP. While its proinflammatory components drive tissue damage in chronic disease, SASP factors also contribute to wound healing, immune surveillance, and embryonic development. This duality has important implications for therapeutic design. Although senomorphics hold genuine promise as anti-aging interventions, substantial gaps remain between preclinical observations and clinical applicability. Future research must clarify the functions of individual SASP components, identify robust senescence biomarkers, and develop selective compounds with defined dose-response relationships and acceptable safety profiles.

## Introduction

The senomorphic field has accumulated a substantial body of preclinical evidence, yet the translation of this evidence into clinical benefit remains largely unrealized. We argue in this review that the central obstacle is not a lack of mechanistic insight, but rather the absence of tissue-selective delivery strategies, validated senescence biomarkers, and compounds with well-defined dose-response relationships in aged organisms. Without these, even mechanistically compelling agents cannot be rationally advanced to human trials. This review therefore aims not only to catalogue current senomorphic candidates, but to critically evaluate the translational readiness of each class and to propose a framework for prioritizing future research efforts.

### Cellular senescence and senescent cells

Cellular senescence (CS) describes a state of permanent, typically irreversible cell cycle arrest. It is currently recognized as a stress response triggered by diverse stimuli, e.g., telomere attrition, genomic and epigenomic damage, unbalanced mitogenic signals, and tumor suppressor activation (Michaloglou et al., 2005; Collado et al., 2007). The process is highly heterogeneous, affected by genetic, epigenetic, and environmental factors, and occurs across a broad spectrum of somatic cell types. CS is established as one of the 12 hallmarks of aging (Michaloglou et al., 2005) and occupies a genuinely paradoxical position in biology: limited or transient senescence acts as a critical barrier against cancer cell proliferation, whereas its chronic persistence is causally implicated in organismal aging and the pathogenesis of numerous age-related diseases (López-Otín et al., 2023; Campisi, 2000; Borghesan et al., 2020).

A hallmark feature of CS is the senescence-associated secretory phenotype (SASP), a complex pro-inflammatory signaling cascade that reinforces senescence through autocrine and paracrine mechanisms, thereby amplifying the accumulation of senescent cells across tissues (Ohtani, 2022). The burden of senescent cells increases with age due to both elevated induction rates and progressive failure of immune-mediated clearance. Persistent senescence is further exacerbated by chronic tissue stress and low-grade inflammation, collectively contributing to conditions such as osteoarthritis, type 2 diabetes mellitus, atherosclerosis, idiopathic pulmonary fibrosis, and chronic liver disease (Muñoz-Espín and Serrano, 2014; Martel et al., 2020). Despite its well-documented pathological role, CS also performs essential physiological functions. It plays an important role in tumor suppression, embryonic tissue patterning and organogenesis, wound healing, tissue remodelling, and the immune-mediated clearance of damaged cells (Lagoumtzi and Chondrogianni, 2021; Davaapil et al., 2017). This context-dependency is not merely a caveat, it fundamentally shapes how therapeutic interventions targeting the SASP must be designed and evaluated (see Discussion).

*In vitro*, senescent cells demonstrate characteristic morphological changes: they become large, flat, vacuolized, and occasionally multinucleated. *In vivo*, however, their morphology tends to conform to tissue-specific architecture, making identification considerably more challenging (Muñoz-Espín and Serrano, 2014; Hayflick, 1965; Hernandez-Segura et al., 2018). What remains consistent across both contexts is their sustained metabolic activity, which enables the generation and release of a broad array of factors that shape the tissue microenvironment in diverse, not always harmful, ways (Figure 1) (Calcinotto et al., 2019; Acosta et al., 2013; Shay and Roninson, 2004).

**FIGURE 1:**
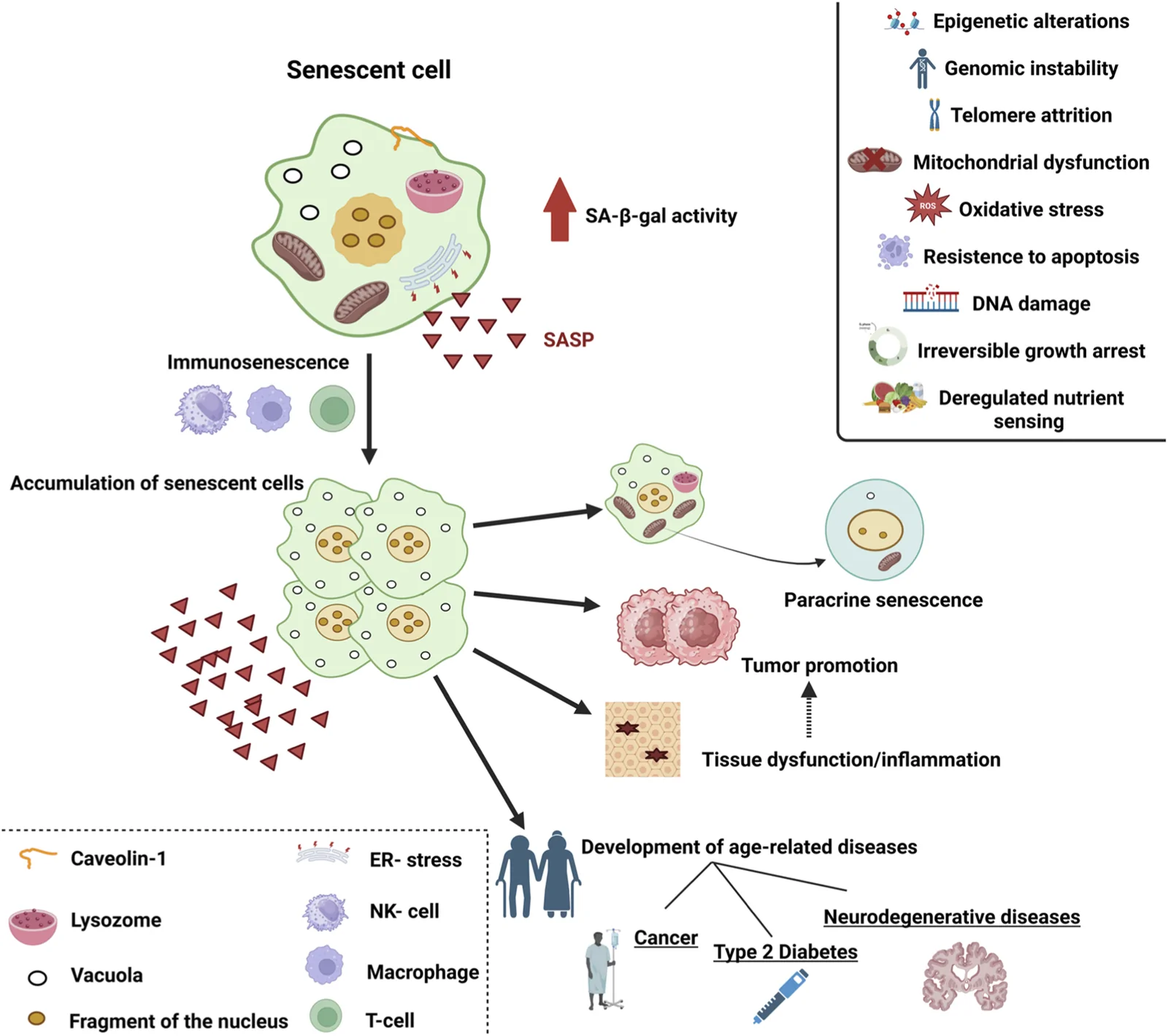
Characteristics and detrimental effects of senescent cells. The figure illustrates nine key features of senescent cells, emphasizing both characteristic morphological changes and the detrimental consequences of their tissue accumulation, largely mediated through the SASP and immunosenescence, i.e., the age-related decline in immune cell function that impairs clearance of senescent cells. Key immune cell types affected include natural killer (NK) cells, macrophages, and T-cells. Compared with proliferating cells, senescent cells typically exhibit increased size, a flattened and vacuolized cytoplasm, and multinucleation partly attributable to loss of the nuclear envelope protein lamin B1. Additional changes include altered plasma membrane composition via caveolin-1 upregulation, accumulation of dysfunctional mitochondria, endoplasmic reticulum (ER) stress, and elevated lysosomal content (Hernandez-Segura et al., 2018). Created with BioRender.com.

### The senescence-associated secretory phenotype

Senescent cells are defined in part by the abundant secretion of a diverse array of proinflammatory mediators into the surrounding tissue microenvironment (Gorgoulis et al., 2019). The SASP encompasses cytokines, chemokines, growth factors, proteases, and non-protein factors such as reactive oxygen species (ROS) and prostaglandins, summarized in Table 1 (Ohtani, 2022; Acosta et al., 2013; Kuilman et al., 2008). SASP factors reinforce the senescent state via autocrine signaling that stabilizes cell cycle arrest (Calcinotto et al., 2019), and simultaneously alter the behaviour of neighbouring non-senescent cells in a paracrine manner, promoting secondary senescence and disrupting tissue homeostasis (Figure 1). The composition and intensity of the SASP are not fixed; they vary considerably depending on the senescence-inducing stimulus, the cell type, and the stage of the senescence program (Lagoumtzi and Chondrogianni, 2021; De Cecco et al., 2019).

**TABLE 1:** SASP classification and prominent representatives (Gorgoulis et al., 2019; Coppé et al., 2010).

Classes of the SASP	Representatives
1. Interleukins (IL)	IL-6, IL-7, IL-1α, IL-1β, IL-13, IL-15
2. Chemokines	IL-8, MIP-1*α*, MIP-3*α*, LEC, Eotaxin, Eotaxin-3, TECK, ENA-78, I-309, I-TAC, GRO-*α*, GRO-*β*, GRO-*γ*, MCP-2, MCP-4, IP-10, GCP-2, CMK
3. Proteases and regulators	TIMP-1, TIMP-2, Cathepsin B, MMP-1, MMP-3, MMP-10, MMP-12, MMP-13, MMP-14, PAI-1, PAI-2, tPA, UPA
4. Growth factors and regulators	bFGF, HGF, KGF, VEGF, GM-CSF, SDF-1, PIGF, NGF, IGFBP-2, IGFBP-3, IGFBP-4, IGFBP-6, IGFBP-7, EGF, Amphiregulin, Epiregulin, Heregulin, Angiogenin
5. Additional proinflammatory factors	IFN-*γ*, BLC, MIF, TGF-*β*, S100A8, S100A9, TNF-α
6. Ligands and receptors	ICAM-1, ICAM-3, sTNFRI, OPG, Fas, UPAR, TRAIL-R3m, sTNFRII, SGP130, EGFR
7. Non-protein soluble factors	PGE2, ROS, NO
8. Insoluble factors	Laminin, Collagens, Fibronectin

### Regulation and function of the SASP

The SASP can be initiated by a range of cellular stressors, including DNA damage, oncogenic mitogenic signals, oxidative stress, and epigenetic alterations (Lagoumtzi and Chondrogianni, 2021; Coppé et al., 2010). Its composition is dynamic and shifts depending on the nature of the inducing stimulus, the cell type involved, and whether senescence is in an early or late phase (Lagoumtzi and Chondrogianni, 2021). Early-phase SASP is characterized by secretion of transforming growth factor-*β* (TGF-*β*), chemokines, and pro-inflammatory cytokines. When TGF-*β* signaling is activated, through either autocrine or paracrine mechanisms, it helps establish and sustain the senescent phenotype (Lagoumtzi and Chondrogianni, 2021; Roberts et al., 1981; Rapisarda et al., 2017). If senescent cells are not cleared promptly and instead accumulate, this early SASP signature persists and transitions to a late-phase program, driven largely by sustained upregulation of the transcriptional regulators nuclear factor kappa B (NF-*κ*B) and CCAAT/enhancer-binding protein *β* (C/EBP*β*) (Lagoumtzi and Chondrogianni, 2021; Chien et al., 2011; Orjalo et al., 2009; Hoare et al., 2016). Elevated C/EBP*β* facilitates late SASP gene expression (Lagoumtzi and Chondrogianni, 2021; Hoare et al., 2016), while NF-*κ*B drives transcription of key proinflammatory mediators, most notably IL-6 and IL-8 (Lagoumtzi and Chondrogianni, 2021; Coppé et al., 2008).

The mechanistic/mammalian target of the rapamycin (mTOR) pathway serves as a central post-transcriptional regulator of the SASP, operating primarily through modulation of NF-*κ*B activity and enhancement of mitogen-activated protein kinase (MAPK)-activated protein kinase 2 (MAPKAPK2) translation (Lagoumtzi and Chondrogianni, 2021; Laberge et al., 2015; Herranz et al., 2015). GATA binding protein 4 (GATA4), another transcription factor implicated in SASP activation, feeds into NF-*κ*B signaling as well (Lagoumtzi and Chondrogianni, 2021; Kang et al., 2015; Rodier et al., 2009). The Janus kinase/signal transducer and activator of transcription (JAK/STAT) cascade regulates expression of numerous proinflammatory SASP components (Lagoumtzi and Chondrogianni, 2021; Yu et al., 2009), and the cyclic GMP-AMP synthase/stimulator of interferon gene (cGAS/STING) pathway has been identified as a critical mediator of the inflammatory SASP, controlling IL-6 and interferon gamma-induced protein 10 (IP-10) secretion via NF-*κ*B in both *in vitro* and *in vivo* models (Glück et al., 2017; Giroud et al., 2023; Schmitz et al., 2023). A detailed description of different signaling pathways regulating the SASP is out of the scope of this review; for more detail, see ref (Giroud et al., 2023; Birch and Gil, 2020). However, their interplay is highly relevant for understanding the pharmacological targets discussed below.

Seen together, the mTOR and NF-*κ*B axes emerge as the most consistently targeted and functionally central regulators of the SASP: mTOR primarily controls the translational machinery underlying SASP production, whereas NF-*κ*B acts as its master transcriptional driver. The JAK/STAT pathway plays a complementary amplifying role, particularly in sustaining cytokine signaling loops. This convergence at multiple regulatory nodes suggests that effective senomorphic strategies may ultimately require combinatorial or multi-target approaches rather than single-pathway blockade.

## Pharmaceutical approaches to SASP regulation

Given the central role of senescent cells in age-related pathology, strategies targeting their elimination (senolytics) or their proinflammatory secretome (senomorphics) have generated substantial interest. This review focuses on senomorphic agents. Senomorphics suppress SASP expression without inducing apoptosis, thereby mitigating the deleterious paracrine effects of senescent cells while preserving their viability. Multiple molecular targets have been identified, including the p38 mitogen-activated protein kinase (p38MAPK), phosphatidylinositol 3-kinase/protein kinase B (PI3K/Akt), mTOR, and JAK/STAT pathways, as well as the transcriptional regulator NF-*κ*B (Luís et al., 2022). For comprehensive coverage of senolytics, the reader is referred to dedicated reviews (Lagoumtzi and Chondrogianni, 2021; Zhang et al., 2023).

### Senomorphics

The following sections highlight the most relevant natural and synthetic senomorphic candidates identified to date. Where possible, we assess not only their mechanisms but also the strength and translational relevance of the supporting evidence. Table 2 provides a structured overview.

**TABLE 2:** Overview of currently identified senomorphic agents and their mechanisms of action.

Class	Compound	Mechanism of senomorphic action	References
Natural compounds			
mTOR inhibitor	Rapamycin	↓ mTORC1; ↑ Nrf2; ↓ NF-*κ*B; ↓ IL-1β translation	(Laberge et al., 2015; Papadopoli et al., 2019; Bjedov and Rallis, 2020; Wang et al., 2017)
SIRT1 activator/NF-*κ*B inhibitor	Resveratrol and STACs	↓ NF-*κ*B; ↑ Nrf2; ↑ SIRT1; ↑ PI3K/Akt (telomerase)	(Liu et al., 2008; Hubbard and Sinclair, 2014; Xu et al., 2018)
NF-*κ*B inhibitor	Apigenin	↓ NF-*κ*B via IRAK1/I*κ*B*α*	(Lim et al., 2015)
NF-*κ*B inhibitor/SIRT1 activator	Kaempferol	↓ NF-κB via IRAK1/I*κ*B*α*; ↑ SIRT1	(Lim et al., 2015; Lee et al., 2019)
Multi-target	EGCG	↓ mTOR; ↑ SIRT1; ↑ SIRT3; ↑ Nrf2	(Han et al., 2012; Lilja et al., 2020; Shin et al., 2016; Kumar et al., 2019)
FDA-approved drugs repurposed as senomorphics			
AMPK activator/NF-*κ*B inhibitor	Metformin	↓ NF-*κ*B (↓ I*κ*B/IKK*α*/*β* phosphorylation); ↓ mitochondrial complex I; ↑ AMPK	(Moiseeva et al., 2013; Algire et al., 2012; OWEN et al., 2000)
JAK/STAT inhibitor	Ruxolitinib	↓ JAK1/2; ↓ STAT3; selective SASP suppression	(Xu et al., 2015a)
Glucocorticoid	Cortisol/Corticosterone	↓ IL-1α/NF-*κ*B positive feedback loop; selective SASP component suppression	(Laberge et al., 2012)
Other senomorphics			
NF-*κ*B inhibitor	SR12343	↓ IKK/NF-*κ*B activation	(Zhang et al., 2021)
NF-*κ*B inhibitor (peptide)	8-NBD peptide	↓ IKK/NF-*κ*B	(Tilstra et al., 2012)
p38MAPK inhibitor	SB203580; UR-13756; BIRB796	↓ p38MAPK; ↓ SASP (IL-6, IL-8, GM-CSF)	(Freund et al., 2011)
MAPKAPK2 inhibitor	MK2.III; PF-3644022	↓ MAPKAPK2; ↓ IL-6	(Alimbetov et al., 2016)
HMG-CoA reductase inhibitor	Atorvastatin; Pravastatin; Pitavastatin; Simvastatin	↑ eNOS; ↑ SIRT1; ↑ catalase via Akt; ↓ proinflammatory SASP	(Liu et al., 2015; Ota et al., 2010)
ATM–HIF1*α*/TRAF-6 blocker	Rutin	× Blocking interactions between ATM and HIF1*α* or TRAF-6; ↓ early and late SASP	(Liu et al., 2024a)
Emerging senomorphic candidates			
ATM inhibitor	KU-60019	↓ ATM kinase; ↓ NF-*κ*B (indirect); ↑ autophagy/lysosome axis	(Kang et al., 2017)
COX inhibitor/SIRT1 modulator	Aspirin	Mechanism on SASP not fully defined; ↓ SA-*β*-gal; ↓ p21; ↑ SIRT1	(Feng et al., 2019)
TGF-*β* pathway modulator	mmu-miR-291a-3p	↓ TGFBR2 signaling	(Bae et al., 2019)
Ca^2+^ channel blocker	Loperamide/Niguldipine	× Ca^2+^ channel blocking; mechanism on SASP not defined	(Fuhrmann-Stroissnigg et al., 2017)
Nrf2 activator/NF-*κ*B inhibitor	Melatonin	↑ Nrf2 via MT1/MT2; ↓ NF-*κ*B	(Fang et al., 2018)
mTOR/JAK/STAT3 inhibitor	Regorafenib	↓ mTORC1 and mTORC2; ↓ JAK/STAT3; ↓ IL-6, IL-8	(Park et al., 2023)
Niacinamide- antioxidant, anti-inflammatory compound Hyaluronic acid- humectant, moisturizer	Niacinamide & Hyaluronic acid	Mechanism on SASP not fully defined; ↓ MMP12, CMK, S100A8, S100A9	(Bogdanowicz et al., 2024)
NF-κB and p38 MAPK modulator	Procyanidin C1 (PCC1)	↓ NF-κB and p38MAPK; ↓ SASP (IL-6, IL-1β, and TNF-α)	(Liu et al., 2024b)
PRDX2-targeting compound	Dihydromyricetin (DMY)	↓ ATM/p38/Akt/mTOR and NF-κB modulation	(Xu et al., 2026)
HSPA8-targeting compound	Pyrroloquinoline quinone (PQQ)	↓ p38/Akt/mTOR and NF-κB modulation	(Jiang et al., 2025)
Unknown target	Enrofloxacin-derived compound C36	Mechanism on SASP not fully defined; ↓ renal p21, p16, and p53	(Chen et al., 2026)
TNF-α and NF-κB modulator	Royal jelly (RJ)	↓ TNF-α and NF-κB	(Nakagawa et al., 2026)
cGAS–STING modulator	Urolithin A (UA)	↓ cGAS–STING	(Barkovskaya et al., 2025)
Broad-spectrum metalloprotease inhibitor	Batimastat (BB-94)	↓ ADAM19; ↓ SASP (IL-6, IL-1α, IL-8); ↓ p21	(Bar et al., 2025)

Arrows indicate direction of regulation (↓ inhibition/decrease; ↑ activation/increase; × selective blocking of interactions). Compound classes are defined at the level of primary cellular target where possible.

### Rapamycin

Rapamycin (sirolimus) is a natural macrolide first isolated from *Streptomyces hygroscopicus* (Vézina et al., 1975), initially characterized as an antifungal agent. Its immunosuppressive (Menicacci et al., 2017) and antiproliferative (Menicacci et al., 2019; Liu et al., 2018) properties subsequently led to FDA approval for prevention of renal transplant rejection and treatment of lymphangioleiomyomatosis (Zhang et al., 2023). Over the past decade, rapamycin has become one of the best-characterized senomorphic agents.

Its senomorphic mechanism involves the inhibition of mTOR complex 1 (mTORC1), which reduces phosphorylation of downstream effectors S6 kinase (S6K) and 4E-binding protein 1 (4E-BP1) (Zhang et al., 2023; Papadopoli et al., 2019; Bjedov and Rallis, 2020), thereby impairing the translational machinery required for SASP production. More specifically, rapamycin reduces IL-6 and IL-8 expression in senescent cells partly by blocking the translation of IL-1β, an upstream activator of NF-*κ*B signaling (Laberge et al., 2015). Secondary effects include activation of the nuclear factor erythroid 2–related factor 2 (Nrf2) pathway (Zhang et al., 2023; Wang et al., 2017) and suppression of NF-*κ*B-mediated IL-1α production (Laberge et al., 2015; Zhang et al., 2023). These findings from preclinical studies provide mechanistic evidence that mTORC1 inhibition can attenuate SASP production and support the senomorphic effects of rapamycin.

Despite encouraging preclinical results, rapamycin carries a significant adverse effect burden, i.e., metabolic dysregulation, thrombocytopenia, hyperlipidemia, and impaired wound healing, attributable at least in part to off-target mTOR complex 2 (mTORC2) inhibition (Zhang et al., 2023; Li et al., 2014). Preclinical work suggests that intermittent low-dose regimens may reduce this toxicity while preserving senomorphic efficacy (Popovich et al., 2014), though the optimal dosing window for human use remains to be established. Analogs (rapalogs) such as everolimus, temsirolimus, and ridaforolimus are under investigation for improved pharmacokinetic and safety profiles (Zhang et al., 2023; Schreiber et al., 2019), but whether they fully replicate rapamycin’s senomorphic effects is not yet clear. Importantly, rapamycin has recently been evaluated in several clinical trials, including the Phase II PEARL study and the RAPA-EX-01 randomized trial in older adults (Moel et al., 2025; Stanfield et al., 2026).

### Resveratrol and sirtuin-activating compounds (STACs)

Resveratrol is a naturally occurring polyphenol found predominantly in red wine and certain fruits. It is widely recognized for its anti-inflammatory (Kopp, 1998) and antioxidant properties (Frankel et al., 1993). Evidence supporting the senomorphic activity of resveratrol has emerged from both *in vitro* models, including Medical Research Council cell strain 5 (MRC-5) fibroblasts (Menicacci et al., 2017; Menicacci et al., 2019), and *in vivo* in the annual fish *Nothobranchius guentheri* (Lagoumtzi and Chondrogianni, 2021; Liu et al., 2018). Mechanistically, resveratrol inhibits NF-*κ*B signaling (Lilja et al., 2020) and activates Nrf2, a negative regulator of NF-*κ*B, reinforcing anti-inflammatory effects (Liu et al., 2008). In senescence-accelerated mouse-prone 8 (SAMP8) mice, an established model of accelerated aging, resveratrol reduced proinflammatory and prooxidative markers in pancreatic tissue through NF-*κ*B downregulation (Lagoumtzi and Chondrogianni, 2021; Farkhondeh et al., 2019).

A key molecular target is silent information regulator 2-related enzyme 1 (SIRT1), a NAD^+^-dependent deacetylase whose expression declines with age and whose activation has been shown to delay senescence across multiple model organisms (Zhang et al., 2023; Chen et al., 2020). Resveratrol exhibits a biphasic dose-response via SIRT1: at low concentrations (generally <10 μM) it functions as a senomorphic agent or antioxidant, inhibiting endothelial progenitor cell senescence via PI3K-Akt-dependent telomerase activation (Zhang et al., 2023; Xia et al., 2008) and attenuating neuronal senescence via SIRT1-dependent STAT3 modulation (Zhang et al., 2023; Liu et al., 2021). At higher concentrations (>25 μM), however, it becomes pro-oxidant and can induce growth arrest, senescence, or apoptosis (Zhang et al., 2023; Shaito et al., 2020). Such biphasic behavior underscores the importance of precise dosing for therapeutic applications.

Despite these promising findings, the translational relevance of resveratrol remains contested. A substantial proportion of reported effects derives from *in vitro* or short-lived animal models, while clinical outcomes in humans have been inconsistent. Major limiting factors include poor oral bioavailability, rapid first-pass metabolism, and the need for supraphysiological concentrations to achieve meaningful senomorphic effects, i.e., concentrations that may be difficult or impractical to sustain clinically. These limitations have driven the development of second-generation STACs (SRT1720, SRT1460, SRT2183) and third-generation agents (STAC-5, STAC-9, STAC-10), which aim to overcome the pharmacological shortcomings of the parent compound (Zhang et al., 2023; Hubbard and Sinclair, 2014). Whether these analogs will deliver more consistent clinical outcomes remains to be determined.

### Apigenin and kaempferol

Apigenin and kaempferol are flavonoids widely distributed in fruits, vegetables, herbs, and green tea. Both inhibit NF-*κ*B signaling via suppression of the interleukin-1 receptor-associated kinase one/inhibitor of nuclear factor kappa B alpha (IRAK1/I*κ*B*α*) pathway. In bleomycin-induced senescent BJ fibroblasts, these compounds effectively reduced SASP expression (Lagoumtzi and Chondrogianni, 2021; Lim et al., 2015), and oral apigenin significantly lowered SASP factor levels in kidneys of aged rats, indicating systemic senomorphic potential.

Three kaempferol-type compounds isolated from rambutan (*Nephelium lappaceum*) seeds were found to downregulate mRNA expression of IL-6, IL-8, IL-1α, and IL-1β, while simultaneously upregulating SIRT1 (Lee et al., 2019; Lee et al., 2020), suggesting a dual mechanism of SASP suppression and longevity pathway activation. The evidence base, however, remains primarily preclinical, and bioavailability data for these specific compounds are limited.

### Epigallocatechin gallate (EGCG)

EGCG is the principal bioactive polyphenol in green tea and is well-recognized for its antioxidant and anti-inflammatory properties (Lagoumtzi and Chondrogianni, 2021; Farkhondeh et al., 2019). Its potential relevance to aging biology stems from pleiotropic effects on multiple senescence-associated pathways: mTOR inhibition (White, 2019), activation of SIRT1 (Han et al., 2012) and SIRT3 (Lilja et al., 2020), the latter linked to improved mitochondrial function, and Nrf2 pathway induction with upregulation of cytoprotective genes (Shin et al., 2016).

The ability of EGCG to modulate multiple signaling pathways suggests that it may act as a multi-target senomorphic agent. It is worth noting, however, that the evidence base consists almost entirely of *in vitro* and short-term animal studies. The bioavailability of EGCG after oral administration is limited and variable, and whether the concentrations required to modulate the pathways listed above are achievable in relevant human tissues has not been systematically addressed.

### Metformin

Metformin is a biguanide prescribed as first-line therapy for type 2 diabetes mellitus and is among the most clinically advanced candidates for senomorphic therapy. Its proposed senomorphic effects involve at least three mechanistic axes: (i) suppression of NF-*κ*B through inhibition of I*κ*B and inhibitor of nuclear factor kappa-B kinase subunit alpha and beta (IKK*α*/*β*) phosphorylation, reducing SASP components including IL-1β, IL-6, IL-8, and epithelial-derived neutrophil-activating peptide 78 (ENA-78) in senescent IMR90 fibroblasts (Lagoumtzi and Chondrogianni, 2021; Zhang et al., 2023; Moiseeva et al., 2013); (ii) inhibition of mitochondrial complex I, reducing ROS production and oxidative DNA damage, factors directly associated with CS induction (Lagoumtzi and Chondrogianni, 2021; Algire et al., 2012; OWEN et al., 2000); and (iii) the adenosine monophosphate-activated protein kinase (AMPK) pathway activation, with tissue-specific benefits demonstrated in senescent lens epithelial cells of aged mice (Chen et al., 2022).

Metformin has extended lifespan and healthspan in both mice (Martin-Montalvo et al., 2013) and *Caenorhabditis elegans* (Cabreiro et al., 2013). Importantly, it is currently the subject of the TAME (Targeting Aging with Metformin) trial, the first major clinical study designed specifically to evaluate a pharmaceutical agent for age-modifying effects in humans. The TAME trial has not yet started enrolling participants and remains unfunded (Zhang et al., 2023; Barzilai et al., 2016). Several clinical trials have investigated the potential senomorphic and geroprotective effects of metformin, including the randomised MET-PREVENT and Phase II METFORAGING trials (Witham et al., 2025; Marcelo-Calvo et al., 2026). Recent clinical studies have provided preliminary evidence that metformin may modulate cellular senescence and SASP in humans, although its clinical relevance remains uncertain. Further clinical studies are needed to determine whether these biological effects translate into meaningful improvements in healthspan and aging-related outcomes.

Epidemiological data are intriguing: metformin users with type 2 diabetes show lower all-cause mortality and reduced incidence of age-related diseases compared with non-diabetic controls not taking the drug (Zhang et al., 2023; Bannister et al., 2014). Whether this reflects direct SASP modulation, broader metabolic or anti-inflammatory effects, or patient selection bias remains to be clarified in adequately powered prospective studies. Metformin’s precise mechanism of action remains incompletely defined even after decades of clinical use, a reminder that mechanistic clarity and clinical efficacy do not always go hand in hand.

### JAK/STAT inhibitors

The JAK/STAT pathway plays a pivotal role in sustaining proinflammatory SASP expression. JAK kinase activation leads to STAT3 phosphorylation, which modulates B Cell lymphoma 2 (Bcl-2) family proteins, upregulates receptors for IL-6 and IL-8, and maintains NF-*κ*B activation during prolonged inflammatory states (Kunigal et al., 2009; Wang et al., 2012; Liang et al., 2013). Pharmacological JAK1/2 inhibition, with ruxolitinib as the most studied compound, reduces secretion of granulocyte-macrophage colony-stimulating factor (GM-CSF), growth-regulated oncogene-alpha (GRO-*α*), IL-6, IL-8, and other proinflammatory SASP factors. This effect was associated with reduced systemic and adipose tissue inflammation and improved physical performance in aged mice. Notably, certain components such as IL-1α, IL-1β, IL-7, interferon-gamma (IFN-*γ*), epidermal growth factor (EGF), and basic fibroblast growth factor (bFGF) appear largely unaffected (Xu et al., 2015a), reflecting the selective rather than comprehensive nature of JAK-targeted SASP modulation.

Ruxolitinib, FDA-approved drug for myelofibrosis (Lagoumtzi and Chondrogianni, 2021), prevented age-related adipose tissue loss, reduced lipotoxicity, improved insulin sensitivity, and enhanced physical performance in 22-month-old mice (Xu et al., 2015a; Xu et al., 2015b). Collectively, these *in vitro* and *in vivo* findings provide preclinical support for the potential senomorphic effects of ruxolitinib. The major clinical concern, however, is the well-documented risk of opportunistic infections, including Herpes zoster, associated with JAK inhibition. This risk is particularly relevant in older patients who already experience age-related immune decline, complicating the benefit-risk calculation for chronic senomorphic use (Romashkan et al., 2021; Winthrop, 2017).

### Glucocorticoids (GCs)

In an *in vitro* study, endogenous GCs such as cortisol and corticosterone selectively suppressed certain SASP components, notably IL-6, IL-8, GM-CSF, and monocyte chemotactic protein-2 (MCP-2), by disrupting the IL-1α/NF-κB positive feedback loop. This selectivity is worth emphasizing, since GCs are not broad SASP suppressors, and their effect profile differs from that of compounds like rapamycin or metformin. GCs can also attenuate the capacity of the SASP to promote tumor cell invasion, suggesting a potential role in mitigating paracrine oncogenic effects of senescent cells (Lagoumtzi and Chondrogianni, 2021; Laberge et al., 2012).

The therapeutic utility of GCs for senomorphic purposes is significantly constrained by their adverse effect profile with long-term use, i.e., osteoporosis, muscle atrophy, skin thinning, metabolic dysregulation, which would likely be unacceptable in aging populations where these risks are already elevated (Le et al., 2022). These limitations underscore the general principle that repurposing existing anti-inflammatory drugs as senomorphics requires careful evaluation of whether the pharmacological safety margin is compatible with chronic administration in older, often frail individuals.

### NF-*κ*B inhibitors

Given NF-*κ*B’s central role in SASP regulation, its pharmacological targeting has attracted considerable interest. An *in vivo* study showed that the 8-NEMO-binding domain (8-NBD) peptide, which selectively inhibits the I*κ*B kinase (IKK)/NF-*κ*B axis, reduced CS and ameliorated age-associated pathologies in *Ercc1* ^*–/Δ*^ progeroid mice. Detailed microarray analysis revealed modulation of IL-6 and IL-8 signaling pathways (Park and Shin, 2022; Tilstra et al., 2012). However, the inherent limitations of peptide-based approaches, i.e., poor metabolic stability and low bioavailability, have motivated the development of small-molecule alternatives.

SR12343, a selective small-molecule IKK/NF-κB inhibitor, decreased CS markers and SASP components (IL-1α, IL-1β, IL-6) in fibroblasts under genotoxic and oxidative stress. *In vivo*, it reduced senescence burden, improved tissue pathology, and enhanced healthspan in both naturally aged mice and accelerated aging models (Zhang et al., 2023; Zhang et al., 2021). These results are encouraging, though it should be noted that all available data are preclinical, and the therapeutic window of systemic NF-*κ*B inhibition would need careful characterization in human subjects. NF-*κ*B signaling intersects with insulin-like growth factor 1 (IGF-1), mTOR, sirtuins, Forkhead box O3, subclass a (FOXO3a), Nrf2, and the DNA damage response (DDR) (Zhang et al., 2019), making it a highly connected but also pharmacologically complex target.

### p38MAPK inhibitors

Based on *in vitro* findings, p38MAPK has emerged as a regulator of the SASP that operates predominantly independently of DNA damage signaling, i.e., mechanistically distinct entry point compared with NF-*κ*B or mTOR inhibitors (Freund et al., 2011). Selective inhibition with SB203580 substantially reduced secretion of IL-6, IL-8, and GM-CSF and attenuated paracrine effects of senescent cells induced by ionizing radiation or oncogenic Ras expression (Zhang et al., 2023; Freund et al., 2011). Similar results were obtained with UR13756 and BIRB796 (Alimbetov et al., 2016). Downstream, MAPKAPK2 effector has been identified as a critical regulator of SASP expression. Small molecule inhibitors targeting MAPKAPK2, MK2. III and PF-3644022, reduced IL-6 secretion in senescent cells (Zhang et al., 2023; Alimbetov et al., 2016).

This body of work, while mechanistically coherent and consistently replicated across multiple inhibitors, remains exclusively preclinical. The challenge of achieving selective p38MAPK inhibition without compromising its broader physiological roles, including responses to cellular stress, remains a concern for clinical development.

### Statins

Statins (HMG-CoA reductase inhibitors) represent an interesting class in the senomorphic context because of their already widespread clinical use and established safety profile. In senescent human fibroblasts, simvastatin suppressed the cancer-promoting effects of the SASP on breast cancer cells by reducing IL-1α, IL-1β, IL-6, IL-8, and GRO-*α*. The senomorphic mechanism appears to involve Akt activation at Ser473, leading to upregulation of endothelial nitric oxide synthase (eNOS), SIRT1, and catalase, i.e., molecules associated with improved oxidative stress handling (Zhang et al., 2023; Liu et al., 2015). Other statins, atorvastatin, pravastatin, and pitavastatin, have demonstrated similar SASP-suppressing properties (Ota et al., 2010).

The practical advantage of statins is that their safety profile at standard clinical doses is well-characterized. Although adverse effects such as hepatotoxicity and myopathy, as well as a modestly increased risk of type 2 diabetes, are well documented, they are generally considered manageable and acceptable in current clinical practice (Zhang et al., 2023; Liu et al., 2015). Whether statin doses sufficient to produce meaningful SASP modulation in aged tissues overlap with standard lipid-lowering doses is an open question that warrants dedicated investigation.

### Rutin

Preclinical studies have showed that rutin is a natural bioflavonoid glycoside with antioxidant and anti-inflammatory activity (Liu et al., 2024a; Panasiak et al., 1989). At 100 μM, the most efficacious concentration tested, rutin suppressed a defined set of SASP components including IL-6, IL-8, IL-1α, IL-1β, GM-CSF, matrix metallopeptidase-3 (MMP-3), and GRO-*γ*. Its mechanism involves selective disruption of ataxia-telangiectasia mutated kinase (ATM) interactions with hypoxia-inducible factor 1-*α* (HIF1-*α*) (relevant to early SASP) and with tumor necrosis factor receptor-associated factor 6 (TRAF-6) (a key axis in late SASP development) (Liu et al., 2024a). Subchronic toxicity studies of enzymatically decomposed rutin confirmed an acceptable safety profile (Hasumura et al., 2004). Major limitations include low water solubility and restricted bioavailability, which would need to be addressed before clinical evaluation.

### Emerging and less-characterized senomorphic candidates

A number of additional natural and synthetic compounds have demonstrated anti-aging or anti-senescent properties, although their evidence base is more limited and, in many cases, SASP modulation has not been directly assessed. These candidates are highlighted below as possible areas of emerging interest.

### ATM inhibitors

ATM kinase is activated by DNA damage and sustains the DDR that drives SASP expression (Zhang et al., 2023). KU-60019, identified in a chemical screen, mitigated senescence and accelerated wound healing in aged mice, likely via restoration of the lysosome/autophagy axis and mitochondrial function (Lagoumtzi and Chondrogianni, 2021; Kang et al., 2017; Kim and Kim, 2019; Kuk et al., 2019). SASP components were not directly measured, but the mechanistic link between DDR attenuation and reduced NF-*κ*B activity makes ATM inhibition a plausible senomorphic strategy (Kim and Kim, 2019). This study provides *in vivo* evidence supporting the potential senomorphic effects of ATM inhibition, although direct evidence of SASP modulation remains limited. A critical caveat: prolonged ATM inhibition may compromise genome surveillance and increase cancer risk, i.e., a concern that fundamentally limits chronic therapeutic use (Zhang et al., 2023).

### Aspirin

At low doses, aspirin can inhibit senescence and SASP markers in preclinical models (Hybiak et al., 2020). In a mouse model of chemotherapy-induced childhood senescence, aspirin reduced senescence marker expression and attenuated long-term doxorubicin toxicity (Zhang et al., 2023; Feng et al., 2019). In Alzheimer’s-related neuronal cells, aspirin decreased senescence-associated *β*-galactosidase (SA-*β*-gal)-positive cells, p21, and plasminogen activator inhibitor-1 (PAI-1) expression, and attenuated DDR, partially through SIRT1 modulation (Li et al., 2022). Its mechanism of SASP suppression is not fully defined, and clinical evidence specifically targeting SASP is lacking. The attractiveness of aspirin lies in its established chronic-use safety profile at anti-thrombotic doses (75–100 mg), though this does not automatically translate to efficacy at concentrations needed for SASP modulation.

### Embryonic stem cells (ESCs) and the conditioned medium (CM)

Preclinical evidence suggests that factors derived from ESC-CM, including miRNAs such as mmu-miR-291a-3p, may modulate cellular senescence. Administration of mmu-miR-291a-3p, which inhibits the transforming growth factor beta receptor 2 (TGFBR2) signaling, accelerated skin wound healing in aged mice (Lagoumtzi and Chondrogianni, 2021; Bae et al., 2019), suggesting modulation of TGF-*β*, a critical early SASP regulator. Evidence remains early-stage.

### Calcium channel blockers (loperamide and niguldipine)

Both compounds suppressed SASP in mouse embryonic fibroblasts (MEFs) (Fuhrmann-Stroissnigg et al., 2017). These *in vitro* findings suggest that loperamide and niguldipine may modulate the SASP. Loperamide’s use at higher-than-therapeutic doses carries significant cardiac and neurological risks (Schifano and Chiappini, 2018; White, 2019; Wu and Juurlink, 2017), which would substantially complicate its repurposing for chronic senomorphic applications.

### Melatonin

Declining melatonin secretion with aging suggests a possible anti-aging role. Based on a preclinical study, melatonin activates Nrf2 via melatonin receptor 1/2 (MT1/MT2) receptors and inhibits NF-*κ*B. In canine adipose mesenchymal stem cells, pretreatment enhanced viability and transplantation outcomes in acute liver injury models, though SASP-specific effects require further characterization (Fang et al., 2018).

### Regorafenib

This multi-kinase inhibitor, FDA-approved drug for hepatocellular carcinoma, suppresses Akt/mTOR and JAK/STAT3 signaling including downstream IL-6 and IL-8, suggesting potential senomorphic activity, particularly in inflammatory conditions associated with CS such as emphysema and osteoarthritis (Park et al., 2023). Adverse effects including hand-foot skin reactions, rash, fatigue, and hypertension (Krishnamoorthy et al., 2015) would need to be weighed carefully against any senomorphic benefit. The available *in vitro* and *in vivo* study suggests that regorafenib may exert senomorphic effects through modulation of inflammatory SASP components.

### Niacinamide & hyaluronic acid

An emerging tissue-specific approach that may have senomorphic potential is a topical skin treatment containing niacinamide and hyaluronic acid. In a 2024 clinical study, 2 months of treatment improved several features of skin aging and reduced the expression of genes associated with cellular senescence, including MMP12, CMK, S100A8, and S100A9, suggesting a senomorphic effect in human skin. However, the underlying mechanism is still not fully understood, and evidence is limited by the skin-specific application, short treatment duration, and limited long-term safety data (Bogdanowicz et al., 2024).

### Procyanidin C1 (PCC1)

PCC1 has emerged as a potential senotherapeutic approach for retinal aging. This polyphenolic compound has demonstrated both senolytic and senomorphic effects. In a 2024 preclinical study, PCC1 reduced senescence and SASP-associated cytokines, including IL-6, IL-1β, and TNF-α, while improving retinal structure and function in aged mice. Mechanistically, PCC1 can exert its senomorphic effects partly through suppression of NF-κB and p38MAPK signaling, both of which are key regulators of the SASP (Liu et al., 2024b).

### Dihydromyricetin (DMY)

This natural flavonoid represents a promising senomorphic and senolytic agent. A 2026 preclinical study found that DMY reduced SASP factors such as IL-6, IL-1β, MMP1, and MMP3 by targeting PRDX2, enhancing DNA repair and reducing oxidative stress in senescent fibroblasts. These effects were associated with suppressed activity of the ATM/p38/Akt/mTOR and NF-κB signaling pathways, which are involved in the regulation of the SASP. In senescent microglia, however, DMY disrupted PRDX2-dependent redox balance, causing mitochondrial dysfunction and apoptosis. These effects reduced tissue aging and improved cognition in mice (Xu et al., 2026).

### Pyrroloquinoline quinone (PQQ)

PQQ has emerged as a potential candidate for senomorphic intervention. It is a redox-active compound that may target the pro-inflammatory phenotype of senescent cells. In a 2025 preclinical study, PQQ suppressed the expression of multiple SASP factors, including IL-6, IL-8, IL-1α, IL-1β, GM-CSF, GRO-α, and GRO-γ, while preserving the viability of senescent cells. Mechanistically, PQQ was shown to target the intracellular protein HSPA8, thereby reducing the activity of downstream signaling pathways, including the p38/Akt/mTOR and NF-κB pathways, which are involved in the regulation of SASP expression. In aged mice, PQQ reduced SASP expression and alleviated age-related tissue degeneration (Jiang et al., 2025).

### Enrofloxacin-derived compound C36

C36 is a novel quinoline-based compound derived through structural optimization of the non-antibacterial enrofloxacin derivative C1 and has been investigated for its potential senomorphic activity. C36 showed improved mammalian safety while extending lifespan and healthspan in *C. elegans*. In cellular and mouse models, C36 attenuated cellular senescence and reduced the expression of SASP components, including IL-1β, TNF-α, and MMP-13. In a mouse model of chronic kidney disease, C36 improved renal function and reduced renal fibrosis by approximately 50%, accompanied by decreased renal expression of the senescence-associated markers p21, p16, and p53. These *in vitro* and *in vivo* findings identify C36 as a promising preclinical senomorphic candidate for targeting age-related renal dysfunction (Chen et al., 2026). The underlying mechanism of the senomorphic effect of this compound has not yet been fully understood.

### Royal jelly (RJ)

RJ has been investigated for its potential natural senomorphic activity *in vitro*. RJ suppressed SASP in senescent human epidermal keratinocytes, thereby reducing levels of IL-6, IL-8, IP-10, GRO-α, GRO-β and PAI-1 without inducing cytotoxicity. These effects were associated with suppressed activity of the TNF-α/NF-κB signaling pathway and decreased oxidative stress (Nakagawa et al., 2026).

### Urolithin A (UA)

This compound has emerged as a promising candidate with potential senomorphic activity through its effects on both pro-inflammatory SASP and DAMP (damage-associated molecular patterns) signaling pathways. DAMPs are endogenous molecules released or exposed by stressed, damaged, or senescent cells that act as danger signals and can activate innate immune and inflammatory responses. In senescent fibroblasts, UA reduced the expression and secretion of IL-6, IL-8 and IL-1α, while also limiting the accumulation of cytosolic DNA and the activation of the cGAS–STING signaling pathway (Barkovskaya et al., 2025). Based on the findings of this *in vitro* study, UA may modulate senescence-associated inflammation by intervening in the DAMP signaling pathway at an early stage, rather than by directly inhibiting individual SASP factors, which highlights its potential as a novel senomorphic strategy.

### Batimastat (BB-94)

Another potential senomorphic strategy involves targeting *ADAM19*, a membrane-bound metalloprotease that may contribute to the regulation of the pro-inflammatory SASP. Genome-wide association analysis identified *meltrin*, the *Drosophila* orthologue of mammalian *ADAM19*, as a potential modulator of the SASP. *Meltrin/ADAM19* inhibition reduced DNA damage, SA-β-gal expression, gut permeability, inflammation, and selected SASP factors across *Drosophila*, mouse, and human fibroblast models, suggesting its potential as a senomorphic target. Pharmacological inhibition using BB-94 reduced the expression of pro-inflammatory SASP factors, including IL-6, IL-1α and IL-8, as well as the expression of the senescence marker p21. These *in vitro* and *in vivo* findings support *ADAM19* inhibition as a potential senomorphic approach to mitigating age-related tissue dysfunction through modulation of metalloproteinase-mediated SASP regulation (Bar et al., 2025). The senomorphic mechanisms responsible for these effects have not yet been fully clarified. Although the pharmacological inhibitor BB-94 is not selective for *ADAM19* and may also inhibit other metalloproteases, the similar effects observed following genetic suppression of *ADAM19* support the role of *ADAM19* in the regulation of SASP. The development of selective *ADAM19* inhibitors would therefore be crucial for determining the specific role of *ADAM19* in cellular senescence and the regulation of the SASP, while minimizing side effects and providing a more accurate basis for assessing its therapeutic potential.

Further characterisation of SASP factors contributing to the pro-inflammatory response associated with intestinal permeability and cellular aging may provide important insights into the specific role of *ADAM19* in gut health. Further studies could help to confirm *ADAM19* as a potential biomarker of intestinal integrity and identify it as a promising therapeutic target for the prevention or treatment of gut-related diseases.

### Critical considerations across senomorphic candidates

The selection of described senomorphic candidates includes both well-established candidates extensively discussed in the literature (e.g., rapamycin, metformin, resveratrol, NF-κB inhibitors) and recently identified compounds with emerging senomorphic potential (e.g., PCC1, DMY, C36, RJ). A fundamental limitation running through the entire senomorphic literature is the predominance of preclinical evidence. Many compounds demonstrate convincing SASP modulation *in vitro,* however, these findings frequently fail to translate into consistent or meaningful *in vivo* or clinical outcomes. Differences in pharmacokinetics, tissue distribution, protein binding, metabolic activation, and systemic biological complexity significantly limit extrapolation from cell-based models. Furthermore, the long-term effects of chronic SASP modulation in humans, including potential disruption of beneficial SASP functions, remain poorly understood. Among the compounds reviewed here, metformin and rapamycin have undergone the most extensive clinical evaluation for aging-related outcomes, whereas clinical evidence for other candidates remains limited, short-term, or restricted to specific tissues. This translational gap represents perhaps the central challenge for the field going forward.

### Safety and pharmacology of senomorphic drug treatment

Suppression of the SASP, while therapeutically attractive, requires considerable caution. Senomorphic agents target core cellular signaling pathways (summarized in Figure 2), introducing the possibility of off-target effects that may not become apparent until after prolonged use. Unlike senolytics, which act in discrete, potentially intermittent treatment cycles, senomorphics must be administered chronically to maintain SASP suppression, a requirement that inherently increases cumulative toxicity risk (Lagoumtzi and Chondrogianni, 2021).

**FIGURE 2:**
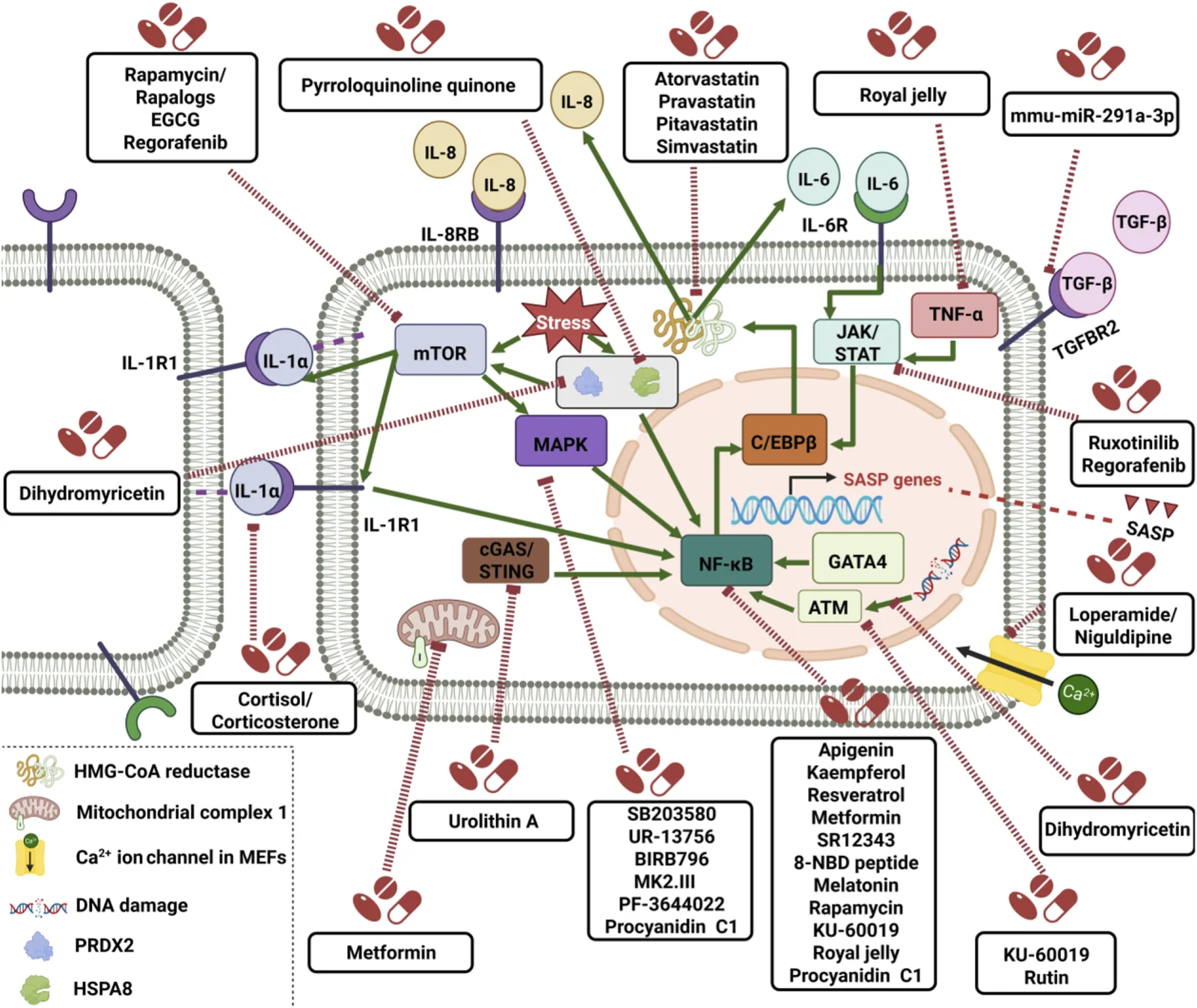
Signaling pathways crucial for SASP regulation and their senomorphic inhibitors/modulators. The figure summarizes all key intracellular pathways involved in SASP regulation and their interconnections (green arrows), as well as the points of inhibition/modulation (red lines) by the prominent senomorphic agents reviewed here. Adapted from (Soto-Gamez and Demaria, 2017). *Created with* BioRender.com.

An important and underappreciated concern is that SASP suppression is not uniformly beneficial. As discussed above, SASP factors contribute to tissue repair, wound healing, immune surveillance, and embryonic development. Indiscriminate or sustained suppression of the SASP risks disrupting these physiological processes. For instance, continuous use of SASP inhibitors may impair cytokine secretion that non-senescent immune cells depend on, potentially compromising innate and adaptive immune responses. This concern is particularly acute for JAK inhibitors, given their established immunosuppressive effects and associated infection risk (Romashkan et al., 2021; Winthrop, 2017), and for rapamycin, which can exacerbate susceptibility to infections at higher doses (Chaib et al., 2022; Neff et al., 2013).

Recent clinical trials have provided further insights into the safety and therapeutic potential of senomorphic agents. In the 48-week Phase II PEARL study, intermittent administration of low-dose rapamycin in healthy older adults was generally well tolerated and was associated with improvements in selected healthspan-related outcomes, although the primary endpoint was not significantly affected (Moel et al., 2025). Similarly, the randomised RAPA-EX-01 study evaluated 13 weeks of rapamycin treatment in older adults undergoing physical training but found no additional functional benefit and reported a higher incidence of adverse effects (Stanfield et al., 2026). Collectively, these findings demonstrate the variability of clinical responses to rapamycin and highlight the importance of optimising treatment regimens and establishing a favourable benefit–risk ratio before its broader clinical application as a potential senomorphic intervention.

Despite a short administration period, rapamycin can exert prolonged senomorphic effects. This may partly reflect inhibition of SASP-mediated propagation of senescence, allowing immune-mediated clearance to gradually reduce the senescent cell burden. Compared with intermittently-administered senolytics, chronic SASP inhibition potentially carries a higher cumulative risk, and this balance between efficacy and safety deserves more systematic investigation than it has received (Lagoumtzi and Chondrogianni, 2021).

Additionally, the randomised MET-PREVENT trial found no improvement in physical performance in older adults with sarcopenia and frailty following 4 months of metformin treatment. A higher incidence of adverse events and hospitalisations was observed in the metformin group (Witham et al., 2025). In contrast, the 96-week Phase II METFORAGING trial found metformin to be generally well tolerated in non-diabetic older adults with HIV, with no significant increase in adverse events compared with placebo. However, metformin did not significantly reduce epigenetic age acceleration (Marcelo-Calvo et al., 2026). Taken together, these findings highlight the heterogeneity of clinical responses to rapamycin and metformin and emphasise the need for further studies aimed at verifying their potential senomorphic effects in humans.

Plant-derived senomorphics, resveratrol, apigenin, rutin, melatonin, EGCG, are commercially available as dietary supplements and are generally considered safe at recommended doses. However, the composition and standardization of such supplements is inconsistently regulated across jurisdictions, and the concentrations required for meaningful SASP modulation may not be achievable through dietary intake alone (Martel et al., 2020; Luís et al., 2022).

Regarding the safety of the most recently identified potential senomorphic agents, allergic reactions represent a particular concern with RJ, as its consumption may trigger severe anaphylactic reactions in susceptible individuals (Végh et al., 2024). BB-94 reached Phase I/II clinical trial in patients with malignant ascites, with additional Phase I studies evaluating intrapleural and intraperitoneal administration (Wojtowicz-Praga et al., 1996; Parsons et al., 1997; Macaulay et al., 1999). However, its clinical development was subsequently discontinued due to poor solubility, limited oral bioavailability, and safety concerns. Moreover, as a non-selective metalloproteinase inhibitor, BB-94 may affect multiple metalloproteinases beyond its intended target, increasing the risk of off-target effects and limiting its therapeutic specificity (Chang, 2023). In contrast, urolithin A and PQQ have demonstrated favorable short-term tolerability in human studies, although their long-term safety remains insufficiently characterized (Denk et al., 2025; Shiojima et al., 2024). For C36, PCC1, and DMY, clinical evidence remains insufficient, as C36 and PCC1 are supported predominantly by preclinical studies (Chen et al., 2026; Liu et al., 2024b), while the registered Phase I study of DMY has not yet generated clinical results.

Polypharmacy is a practically important concern. Older individuals, the intended target population for senomorphic therapy, commonly take five or more medications, substantially increasing the risk of pharmacokinetic and pharmacodynamic interactions. Both ruxolitinib and rapamycin are metabolized by CYP3A and are susceptible to interactions with co-administered CYP3A inhibitors or inducers. JAK inhibitors can affect creatinine clearance, with implications for renally cleared co-medications. Observed reductions in hemoglobin and hematocrit with rapamycin are clinically relevant in patients taking anticoagulants or antiplatelets (Romashkan et al., 2021; Kraig et al., 2018).

Biomarker discovery will be central to the clinical translation of senomorphics. Phase II trials will require validated markers of CS that can define target populations, monitor treatment responses, and detect adverse effects early. Currently available markers, SA-*β*-gal activity and CDKN2A (cyclin-dependent kinase inhibitor 2A), lack specificity for all senescent cell populations (Gorgoulis et al., 2019; Romashkan et al., 2021; Dimri et al., 1995). SA-*β*-gal was among the first screening tools for senescence and remains widely used (Muñoz-Espín and Serrano, 2014; Dimri et al., 1995), but a combination of complementary markers across multiple biological levels is increasingly recognized as necessary for reliable phenotype characterization across diverse tissues.

Recent study has identified further potential biomarkers, including IL-23R, CCL5 and CA13. Age-related changes in circulating proteins were investigated in aged *p16-InkAttac* mice following acute treatment with the senotherapeutics venetoclax, navitoclax, fisetin and luteolin, as well as following transgenic removal of senescent cells. These interventions reversed age-related changes in several circulating factors, including IL-23R, CCL5, and CA13. Corresponding changes were also observed in tissues, particularly in the kidneys. Notably, plasma levels of IL-23R also increased with age in humans, highlighting its potential as a translational biomarker of systemic senescent cell burden and as a potential mediator of senescence-associated inter-organ communication (Carver et al., 2025). Furthermore, in humans, *p16_variant 5* was more predictive of response to dasatinib and quercetin (D + Q) than conventional p16 measurements, while a plasma SASP panel showed comparable predictive performance, highlighting the potential of more specific and multimarker approaches for patient selection and treatment-response assessment in clinical trials (Farr et al., 2025).

## Discussion

Senomorphics represent a biologically coherent but clinically immature therapeutic strategy. A core question facing the field is whether suppression of the SASP alone can produce meaningful and durable clinical outcomes, or whether it merely attenuates downstream effects without addressing the underlying biological problem. This concern motivates ongoing interest in combining senomorphics with senolytics, an approach that in principle could provide broader and more durable benefit.

The case for combining senomorphics with senolytics deserves more than a passing mention. In principle, senolytics clear the accumulated burden of senescent cells, while senomorphics suppress the SASP in cells that have not yet been eliminated or that are performing beneficial functions. The two approaches are therefore complementary rather than redundant. Preclinical work in mouse models has demonstrated that sequential or simultaneous treatment with senolytic and senomorphic agents can produce greater improvements in healthspan markers than either approach alone, though the optimal sequencing, dosing intervals, and tissue contexts for such regimens are not yet established. A further consideration is that senolytic clearance of beneficial senescent populations (for example, during wound healing) may need to be temporarily suspended or geographically restricted, making tissue-targeted delivery a shared priority for both drug classes. Future clinical trial design in this space should explicitly account for these interactions rather than evaluating senomorphics in isolation.

The SASP is not simply a pathological bystander. Its context-dependent beneficial roles, in wound repair, tissue remodeling, immune-mediated clearance, and developmental signaling, are well-established (Lujambio et al., 2013; Krizhanovsky et al., 2008; Demaria et al., 2014; Muñoz-Espín et al., 2013; Storer et al., 2013; Mosteiro et al., 2016). Treating the SASP purely as a target for suppression risks underestimating this complexity. The ideal therapeutic window involves attenuating the chronic, low-grade SASP associated with aging and disease while preserving the acute, transient SASP responses that are essential for normal physiology. No current senomorphic achieves this with adequate selectivity, and developing tools to discriminate between these contexts, whether through targeted delivery, selective pathway inhibition, or biomarker-guided dosing, remains a major challenge.

Among the pathways reviewed, mTOR and NF-*κ*B remain the most validated targets. They are consistently modulated across multiple compound classes, their inhibition produces reproducible SASP suppression in diverse models, and both have been investigated clinically (in other indications) with enough depth to partially characterize their toxicity profiles. The JAK/STAT axis plays an important amplifying role but brings additional immunosuppressive concerns. That several structurally distinct compounds, rapamycin, metformin, EGCG, resveratrol, all converge on mTOR and NF-*κ*B to varying degrees suggests these pathways are genuine bottlenecks in SASP regulation, but also that single-target inhibition will rarely achieve complete suppression.

The translational gap in this field is substantial. The overwhelming majority of senomorphic evidence derives from *in vitro* or short-lived animal models. Where human data exist, as for metformin’s epidemiological associations or aspirin’s established clinical profile, the mechanistic link to SASP modulation specifically remains inferential rather than demonstrated. This is not merely a scientific gap; it affects how the risks and benefits of chronic use should be weighed. It is worth distinguishing between compounds that are repurposed from established clinical use (metformin, statins, aspirin, ruxolitinib), where safety data exist and where dose optimization is more tractable, and purely experimental agents with no clinical history. The former group arguably warrants accelerated investigation precisely because the pharmacological unknowns are fewer.

Whether senomorphics will prove to be a genuine therapeutic advance or a mechanistically interesting but clinically marginal strategy will depend on answers to several interrelated questions: Can relevant tissue concentrations be achieved without systemic toxicity? Can the beneficial and detrimental components of the SASP be selectively discriminated? Will SASP modulation translate into measurable improvements in clinically meaningful outcomes, i.e., functional status, disease incidence, healthcare utilization, in adequately powered trials? Recent clinical studies of rapamycin and metformin, together with future trials such as TAME, may provide important insights into these questions and help clarify their potential senomorphic effects in humans.

## Concluding remarks and future perspectives

CS is one of the twelve hallmarks of aging (López-Otín et al., 2013), and its primary effector mechanism, the SASP, is increasingly recognized as a key driver of age-related pathology. This review has described the biology of CS and the SASP, outlined the regulatory machinery that governs SASP expression, and assessed the current landscape of senomorphic agents with respect to their mechanisms, evidence quality, and translational status.

The most important near-term priority for the field is closing the translational gap. This requires rigorous *in vivo* pharmacokinetic characterization of candidate compounds, development of validated senescence biomarkers that can support clinical trial design, and careful attention to dose-response relationships in aged organisms. It also requires honest acknowledgment of the limitations of existing evidence, most of which is preclinical, short-term, and derived from models that imperfectly recapitulate human aging.

Future research should address several specific priorities: 1) single-cell SASP profiling to resolve the cellular heterogeneity of senescence and identify subpopulations most relevant to specific diseases; 2) development of targeted delivery systems that can concentrate senomorphic activity in pathologically senescent tissue while limiting systemic exposure; 3) systematic evaluation of combinatorial strategies that integrate senomorphics with senolytics or immunomodulatory approaches; and 4) mechanistic clarification of the context-specific beneficial functions of the SASP to define the therapeutic boundaries of its suppression.

Optimization of dosing strategies for already-approved drugs with senomorphic potential, particularly intermittent or low-dose regimens, represents a tractable near-term approach. Rapamycin at intermittent low doses and metformin in the planned TAME trial are among the clearest examples of this logic in practice.

Senomorphics represent a biologically plausible but still clinically immature therapeutic strategy. Preclinical data are increasingly compelling, yet the translation to human benefit remains to be demonstrated for most compounds. The path forward will require improved model systems, better biomarkers, and a willingness to conduct the kinds of rigorous, long-term clinical studies that aging biology has historically lacked. Whether senomorphics will become a cornerstone of anti-aging medicine or remain a mechanistically fascinating but practically limited set of tools will ultimately depend on how effectively these scientific and clinical challenges are met.

## Glossary

4E-BP1: 4E-binding protein 1
8-NBD: 8-NEMO-binding domain
*ADAM19*: A Disintegrin And Metalloproteinase 19
AMPK: Adenosine 5′-monophosphate-activated protein kinase
ATM: Ataxia-telangiectasia mutated kinase
BB-94: Batimastat
Bcl-2: B Cell lymphoma 2
bFGF: Basic fibroblast growth factor
CA13: Carbonic Anhydrase 13
CCL5: Chemokine (C-C motif) ligand 5
CDKN2A: cyclin-dependent kinase inhibitor 2A
C/EBP*β*: CCAAT/enhancer-binding protein *β*
cGAS/STING: Cyclic GMP-AMP synthase/stimulator of interferon genes
CM: Conditioned medium
CMK: Chemokine (C-X-C motif) ligand 9
CS: Cellular senescence
DAMP: damage-associated molecular patterns
D + Q: dasatinib and quercetin
DDR: DNA damage response
DMY: Dihydromyricetin
EGCG: Epigallocatechin gallate
EGF: Epidermal growth factor
ENA-78: epithelial-derived neutrophil-activating peptide 78
eNOS: endothelial nitric oxide synthase
ER: Endoplasmic reticulum
ESCs: Embryonic stem cells
FOXO3a: Forkhead box protein O3a
GATA4: GATA-binding protein 4
GCs: Glucocorticoids
GM-CSF: Granulocyte-macrophage colony-stimulating factor
GRO-*α*/-*β*/-*γ*: Chemokine growth-regulated protein *α*/*β*/*γ*
HIF1-*α*: Hypoxia-inducible factor 1-*α*
HMG-CoA: 3-hydroxy-3-methylglutaryl-coenzyme A
IFN-*γ*: Interferon-*γ*
IGF-1: insulin-like growth factor 1
IKK*α*/*β*: Inhibitor of *κ*B kinase *α*/*β*
IL: Interleukin
IP-10: Interferon gamma-induced protein 10
IRAK1/I*κ*B*α*: Interleukin-1 receptor-associated kinase 1/inhibitor of *κ*B*α*
JAK: Janus kinase
MAPK: Mitogen-activated protein kinase
MAPKAPK2: MAPK-activated protein kinase 2
MCP-2/-4: Monocyte chemotactic protein 2/4
MEFs: Mouse embryonic fibroblasts
MMP: Matrix metallopeptidase
MRC-5: Medical Research Council cell strain 5
MT1/2: Melatonin receptor 1/2
mTOR: Mechanistic/mammalian target of rapamycin
mTORC1/2: mTOR complex 1/2
NF-*κ*B: Nuclear factor *κ*-light-chain-enhancer of activated B cells
Nrf2: Nuclear factor erythroid 2-related factor 2
p38MAPK: p38 mitogen-activated protein kinase
PAI-1/-2: Plasminogen activator inhibitor 1/2
PCC1: Procyanidin C1
PI3K/Akt: Phosphatidylinositol 3-kinase/protein kinase B
PQQ: Pyrroloquinoline quinone
PRDX2: Peroxiredoxin-2
RJ: Royal Jelly
ROS: Reactive oxygen species
S6K: S6 kinase
S100A8/9: S100 calcium-binding protein A8/9
SAMP8: Senescence-accelerated mouse-prone 8
SA-*β*-gal: Senescence-associated *β*-galactosidase
SASP: Senescence-associated secretory phenotype
SIRT1: Sirtuin 1
STACs: Sirtuin-activating compounds
STAT: Signal transducer and activator of transcription
TGF-*β*: Transforming growth factor *β*
TGFBR2: TGF-*β* receptor 2
TNF-α: Tumor necrosis factor α
TRAF-6: TNF receptor-associated factor 6
UA: Urolithin A.
